# TyG index is associated with colorectal adenomatous polyps and genetically linked to colon polyps: a cross-sectional and Mendelian randomization study

**DOI:** 10.3389/fonc.2026.1756399

**Published:** 2026-07-08

**Authors:** Yingyi Li, Liangfu Guo, Xiaodong Zhu, Chanchan Lin, Xiaoqiang Liu, Zicheng Huang, Yisen Huang

**Affiliations:** 1 Department of Gastroenterology, The First Hospital of Quanzhou Affiliated to Fujian Medical University, Quanzhou, China; 2 Department of Endoscopic Center, The First Hospital of Quanzhou Affiliated to Fujian Medical University, Quanzhou, China

**Keywords:** colorectal polyps, cross-sectional, insulin resistance, Mendelian randomization, TyG index

## Abstract

**Background:**

The triglyceride-glucose (TyG) index, a simple and validated surrogate marker of insulin resistance, may be clinically relevant to colorectal polyp development because insulin resistance can promote colorectal neoplasia through hyperinsulinemia, chronic inflammation, oxidative stress, and dysregulated insulin-like growth factor signaling. However, the relationship between TyG index and colorectal polyps remains incompletely understood. This study aimed to investigate the association between TyG index and colorectal polyp risk using both observational and genetic approaches.

**Methods:**

We conducted a cross-sectional study of 4,263 patients undergoing colonoscopy. Associations between TyG index and colorectal adenomatous polyps were assessed using multivariable logistic regression. A two-sample Mendelian randomization (MR) analysis evaluated potential causal effects, with inverse-variance weighted (IVW) method as primary analysis and multiple sensitivity analyses.

**Results:**

Although the crude distribution of colorectal polyps across TyG quartiles did not show a strictly monotonic pattern, participants in different TyG quartiles differed substantially in age, sex distribution, BMI, lifestyle factors, comorbidities, and metabolic profiles. Therefore, multivariable logistic regression was performed to account for potential confounding. Higher TyG index was significantly associated with increased prevalence of colorectal adenomatous polyps after full adjustment (odds ratio [OR] 1.13, 95% CI: 1.01–1.27; P-trend = 0.032). Restricted cubic spline analysis showed no evidence of a nonlinear association between the TyG index and colorectal adenomatous polyp risk (P for nonlinearity = 0.459), supporting the use of a linear model in this dataset. MR analysis supported a observational and potential causal association between genetically predicted TyG index and colon polyps (inverse-variance weighted OR: 1.20, 95% CI: 1.10–1.31; P < 0.001), with consistent results across sensitivity analyses and no evidence of directional pleiotropy (P = 0.884). However, no significant association was found for rectal polyps.

**Conclusion:**

Both observational evidence and genetic evidence support a positive association between the TyG index and colon polyps, whereas the MR analysis did not provide evidence for a causal association with rectal polyps. These findings suggest the potential utility of the TyG index in risk stratification and underscore the role of insulin resistance in the pathogenesis of colorectal neoplasia.

## Introduction

Colorectal cancer (CRC) poses a substantial global public health burden, ranking as the third most frequently diagnosed malignancy and the second leading cause of cancer-associated deaths worldwide (1). The adenoma-carcinoma sequence is a well-established pathway, wherein colorectal adenomatous polyps serve as critical precursor lesions for the majority of sporadic CRC cases (2). The detection and removal of these polyps during colonoscopy have proven highly effective in reducing CRC incidence and mortality, underscoring the importance of early intervention (3). Consequently, identifying modifiable risk factors for colorectal polyp development is a pivotal strategy for the primary prevention of CRC.

Beyond established risk factors such as advancing age, male sex, smoking, obesity, and dietary patterns, there is growing recognition of the role of metabolic dysregulation in colorectal carcinogenesis (4–9). Insulin resistance (IR), a core pathophysiological feature of type 2 diabetes and metabolic syndrome, has emerged as a potential key player. Hyperinsulinemia and increased bioactivity of insulin-like growth factor-1 associated with IR may promote cellular proliferation, inhibit apoptosis, and foster a pro-tumorigenic inflammatory microenvironment within the colonic mucosa (10). While epidemiological studies have suggested a link between IR and an increased risk of colorectal neoplasia, findings specifically regarding colorectal polyps have been inconsistent (11, 12). A significant limitation hindering progress in this area is the practical difficulty of applying the gold-standard method for assessing IR—the hyperinsulinemic-euglycemic clamp—in large-scale epidemiological studies due to its complexity, cost, and invasiveness (13).

To overcome this barrier, the triglyceride-glucose (TyG) index, calculated from fasting triglyceride and fasting plasma glucose levels, has been proposed as a simple, inexpensive, and reliable surrogate marker of IR (14). Substantial evidence validates its strong correlation with clamp-measured IR and its utility in predicting incident type 2 diabetes and cardiovascular diseases (15, 16). Recently, several observational studies have begun to explore the association between the TyG index and CRC risk, generally reporting positive correlations (17–21). However, evidence linking the TyG index specifically to the earlier stage of colorectal polyp development remains scarce. A recent cross-sectional study by Xu et al. reported that elevated TyG index was associated with an increased odds of colorectal adenomatous polyps (22), yet such observational designs are inherently limited. Although Xu et al. reported that elevated TyG index was associated with an increased prevalence of colorectal adenomatous polyps, their study was based on an observational design and therefore could not fully address residual confounding or reverse causation. The observed association between TyG index and polyps could be confounded by unmeasured or imperfectly measured factors such as diet, physical activity, or other metabolic parameters. Furthermore, reverse causation remains a possibility, whereby the presence of a polyp or subclinical metabolic changes associated with early neoplasia might influence TyG index levels. Moreover, whether the association between TyG index and colorectal polyps differs by anatomical site remains unclear. Thus, it remains unclear whether the TyG index is a causal risk factor for colorectal polyps or merely an epiphenomenon.

Mendelian randomization (MR) offers a powerful methodological approach to address this question of causality (23). By using genetic variants associated with the exposure (e.g., TyG index) as instrumental variables, MR can estimate the causal effect of the exposure on an outcome while largely avoiding confounding and reverse causation biases, given that genetic alleles are randomly assigned at conception and fixed throughout life (23). A two-sample MR design, leveraging summary-level data from large genome-wide association studies (GWAS), provides a particularly efficient framework for such causal inference (24).

Therefore, to comprehensively investigate the nature of the relationship between TyG index and colorectal polyps, we designed a study that integrates both observational and genetic causal inference approaches. This study aimed to investigate the association between the TyG index and colorectal polyp risk using both cross-sectional and Mendelian randomization approaches, with additional site-specific evaluation of colon and rectal polyps. We hypothesize that a higher TyG index is not only associated with an increased prevalence of colorectal adenomatous polyps in observational analysis but that this relationship reflects an underlying causal association.

## Methods

### Research design

This study utilized a combined cross-sectional study and MR design to thoroughly examine the causal link between the TyG index and colorectal polyps. The study protocol was granted ethical approval by the Institutional Review Board of Quanzhou First Hospital, conducted in accordance with the ethical principles of the Declaration of Helsinki, with a waiver of informed consent obtained. Furthermore, this investigation adhered to the STROBE and STROBE-MR guidelines to ensure methodological rigor and transparency in reporting this observational study and Mendelian randomization analysis.

### Study population

**Figure 1:**
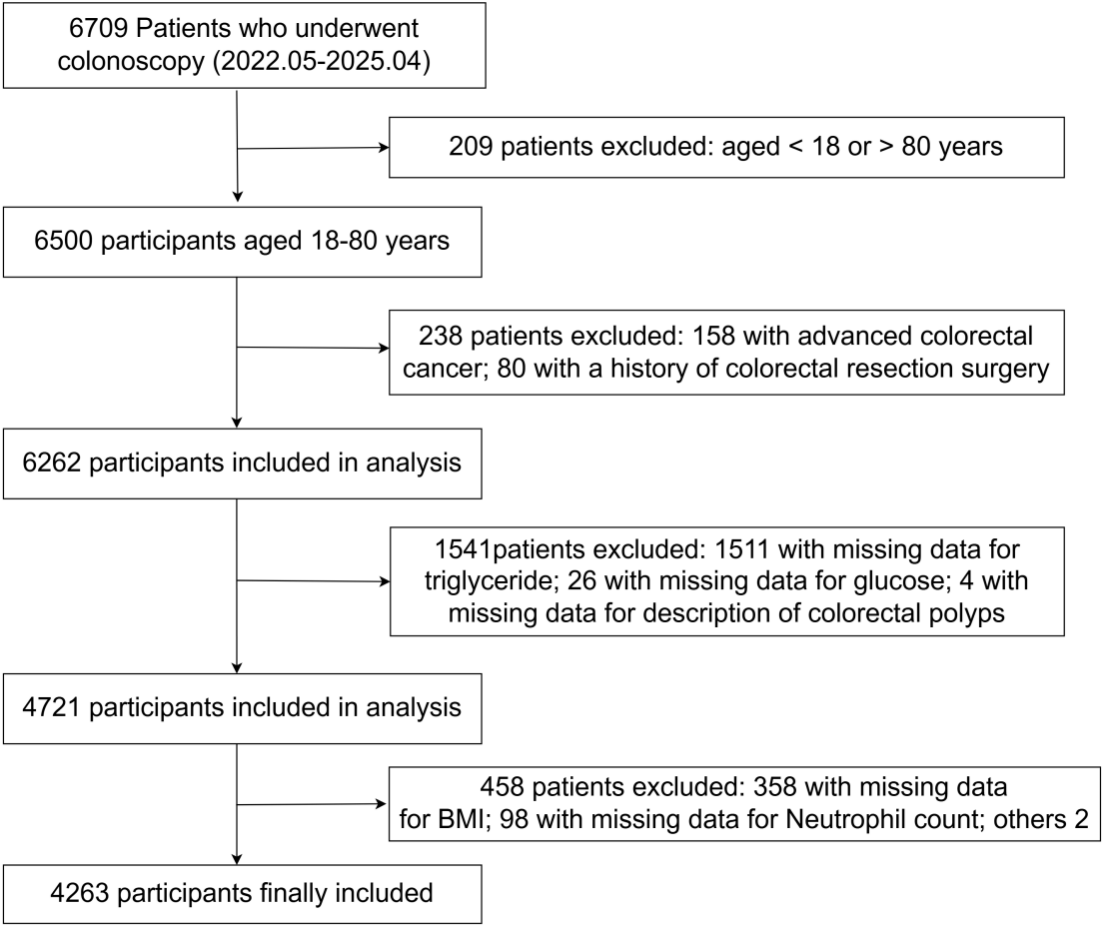
Diagram of patient selection for the study population.

This study retrospectively enrolled patients who underwent colonoscopy at the Department of Gastroenterology, Quanzhou First Hospital between May 1, 2022, and April 30, 2025. The inclusion criteria were: (1) adult patients aged 18–80 years; and (2) completion of a full colonoscopic examination. The exclusion criteria were: (1) advanced colorectal cancer; (2) prior colectomy; (3) missing key variables (triglycerides, glucose, or polyp characterization) (4); subjects lacking essential covariate data. Following the implementation of these selection criteria, a total of 4,263 participants were included in the final analytical cohort (**Figure 1**).

### Assessment of TyG index

Serum triglyceride and fasting blood glucose levels were quantified for all participants using enzymatic colorimetric assays at the clinical laboratory of Quanzhou First Hospital. The TyG index was calculated according to the following formula: TyG index = ln[TG (mg/dL) × FBG (mg/dL)/2]. Participants were stratified into quartiles (Q1-Q4) based on TyG index values to assess the association between TyG index gradients and colorectal polyp occurrence.

### Colorectal polyps ascertainment

All endoscopic procedures were performed by experienced gastroenterologists. Standardized bowel preparation was achieved using 4 liters of polyethylene glycol solution administered the evening prior to colonoscopy. A minimum withdrawal time of 6 minutes was strictly maintained during the procedure, with comprehensive documentation of polyp characteristics including size, number, location and macroscopic features. After the colonoscopy, experienced pathologists conducted histopathological examinations on the polyp specimens to assess the pathological types of the polyps. In the observational analysis, the outcome was colorectal adenomatous polyps confirmed by histopathology. In the MR analysis, publicly available GWAS summary statistics for colon polyps and rectal polyps were used.

### Data collection and definition of covariables

Detailed baseline characteristics were systematically collected for all participants, encompassing gender, age, marital status (married versus unmarried or divorced), body mass index (BMI), systolic and diastolic blood pressure, smoking status (current or former smokers versus never smokers), alcohol consumption (yes/no), and comorbidities including diabetes mellitus, hypertension, and coronary heart disease. Laboratory test results included white blood cell count, neutrophil count, red blood cell count, hemoglobin, platelet count, albumin, aspartate aminotransferase, alanine aminotransferase, gamma-glutamyl transferase, total cholesterol, low-density lipoprotein cholesterol, high-density lipoprotein cholesterol, triglycerides, fasting blood glucose, blood urea nitrogen, creatinine, uric acid, carcinoembryonic antigen (CEA), and carbohydrate antigen 19-9 (CA19-9).

### MR analysis

To evaluate the causal relationship between genetically predicted TyG index and colorectal polyp risk, we employed a two-sample Mendelian randomization approach. Genetic instruments for the TyG index comprised 192 SNPs associated with TyG index derived from a previously published GWAS utilizing the UK Biobank database (**Supplementary Table 1**). The inclusion criteria were set at P < 5 × 10−8, with linkage disequilibrium pruning (r2 < 0.01) applied to ensure independence. Furthermore, SNPs demonstrating significant associations with TG and FBG, as well as those correlated with non-lipid and non-glycemic traits—including systolic blood pressure (SBP), diastolic blood pressure (DBP), and BMI—were excluded to mitigate potential horizontal pleiotropy, using a threshold of P < 1 × 10−7. The colorectal polyps dataset was derived from the US Veterans Affairs Million Veteran Program, encompassing colon polyps (GCST90475319) and rectal polyps (GCST90478441) (details in **Supplementary Table 2**). Mendelian randomization relies on three core assumptions to evaluate causal associations between exposures and outcomes: firstly, the SNPs selected as instrumental variables must exhibit strong associations with the TyG index; secondly, the genetic variants should demonstrate no association with other confounding factors; finally, the influence of genetic variants on colorectal polyps must occur solely through their effect on TyG levels (**Supplementary Figure 1**). We employed five distinct Mendelian randomization approaches to examine the causal relationship between the TyG index and colorectal polyps, including the weighted median method, inverse-variance weighted (IVW) method, simple mode method, weighted mode method, and MR-Egger method. Directional pleiotropy was assessed via the MR-Egger intercept, while heterogeneity among genetic variants was evaluated using Cochran’s Q test.

### Statistical analysis

Continuous variables with normal distribution are presented as mean ± standard deviation, while those with skewed distribution are described as median (interquartile range [IQR]). Categorical variables are expressed as frequency (percentage). We utilized the chi-square test for comparing categorical variables across groups. For continuous variables, one-way ANOVA or Kruskal-Wallis H test was employed to assess differences among TyG quartile groups based on distribution normality, whereas independent Student’s t-test or Mann-Whitney U test was applied to evaluate differences between the colorectal polyps group and the non-colorectal polyps-group. Trend tests were performed by treating the median values of TyG index quartiles as a continuous variable in linear regression models to calculate P-values for trend, thereby validating the results obtained when analyzing the TyG index as a continuous variable. We employed restricted cubic spline models to construct smooth curves for examining potential nonlinear dose-response relationships between the TyG index and colorectal polyps. To ensure the robustness of outcomes, we additionally conducted sensitivity analyses including (1): subgroup analyses stratified by age, sex, BMI, and presence of comorbidities, along with interaction tests (2); multiple imputation with 50 iterations using the chained equations approach to maximize statistical power and minimize potential bias introduced by missing data, followed by repeated logistic regression analyses on the imputed datasets. All analyses were performed using R Statistical Software (Version 4.2.2, http://www.R-project.org, The R Foundation) and Free Statistics analysis platform (Version 1.9, Beijing, China, http://www.clinicalscientists.cn/freestatistics). A two-sided P value < 0.05 was considered statistically significant.

## Results

### Baseline characteristics by TyG quartiles

**Table 1** presents baseline characteristics stratified by TyG index quartiles (n=4263). Significant differences (p<0.001) were observed across quartiles for all variables except coronary heart disease (CHD; p=0.540). Higher TyG quartiles (Q3-Q4) demonstrated progressively greater proportions of males, smokers, drinkers, and participants with hypertension, and diabetes. Similarly, age, BMI, blood pressure (SBP/DBP), most hematological parameters (WBC, NEU, RBC, HGB, PLT), liver enzymes (AST, ALT, GGT), lipid profiles (TG, TC, LDL-C, FBG), renal markers (BUN, CREA, UA), and tumor markers (CEA, CA199) increased significantly across ascending TyG quartiles, while HDL-C decreased (all p<0.001).

**Table 1:** The baseline characteristics stratified by TyG index quartiles.

Characteristic	Total (*n* = 4263)	Q1 (*n* = 1058)	Q2 (*n* = 1048)	Q3 (*n* = 1073)	Q4 (*n* = 1084)	*p*-value
Gender, n (%)						< 0.001
Male	2397 (56.2)	459 (43.4)	545 (52.0)	628 (58.5)	765 (70.6)	
Female	1866 (43.8)	599 (56.6)	503 (48.0)	445 (41.5)	319 (29.4)	
Age, years	53.9 ± 12.5	51.3 ± 14.3	54.8 ± 12.6	55.5 ± 11.5	53.8 ± 11.3	< 0.001
Marital, n (%)						< 0.001
Married	4097 (96.1)	972 (91.9)	1010 (96.4)	1053 (98.1)	1062 (98.0)	
Single	166 (3.9)	86 (8.1)	38 (3.6)	20 (1.9)	22 (2.0)	
BMI, kg/m^2^	23.5 ± 3.5	21.4 ± 3.0	23.2 ± 3.5	24.0 ± 3.2	25.2 ± 3.4	< 0.001
SBP, mmHg	127.0 ± 17.8	120.8 ± 17.0	126.4 ± 17.6	129.1 ± 18.0	131.6 ± 16.9	< 0.001
DBP, mmHg	81.3 ± 11.4	77.3 ± 10.4	80.5 ± 10.9	82.2 ± 11.0	85.0 ± 11.7	< 0.001
Smoking, n (%)						< 0.001
Smoker	865 (20.3)	158 (14.9)	169 (16.1)	238 (22.2)	300 (27.7)	
Non-smoker	3398 (79.7)	900 (85.1)	879 (83.9)	835 (77.8)	784 (72.3)	
Drinking, n (%)						< 0.001
Drinker	694 (16.3)	108 (10.2)	132 (12.6)	191 (17.8)	263 (24.3)	
Non-drinker	3569 (83.7)	950 (89.8)	916 (87.4)	882 (82.2)	821 (75.7)	
Hypertension, n (%)						< 0.001
Yes	1022 (24.0)	147 (13.9)	210 (20.0)	304 (28.3)	361 (33.3)	
No	3241 (76.0)	911 (86.1)	838 (80.0)	769 (71.7)	723 (66.7)	
Diabetes, n (%)						< 0.001
Yes	464 (10.9)	51 (4.8)	73 (7.0)	120 (11.2)	220 (20.3)	
No	3799 (89.1)	1007 (95.2)	975 (93.0)	953 (88.8)	864 (79.7)	
CHD, n (%)						0.540
Yes	130 (3.0)	27 (2.6)	31 (3.0)	39 (3.6)	33 (3.0)	
No	4133 (97.0)	1031 (97.4)	1017 (97.0)	1034 (96.4)	1051 (97.0)	
WBC, ×10^9^/L	6.3 ± 2.1	5.7 ± 1.8	6.2 ± 2.0	6.4 ± 1.9	7.0 ± 2.3	< 0.001
NEU, ×10^9^/L	3.8 ± 1.8	3.4 ± 1.6	3.8 ± 1.7	3.9 ± 1.6	4.2 ± 2.1	< 0.001
RBC, ×10^9^/L	4.6 ± 0.6	4.4 ± 0.6	4.5 ± 0.6	4.7 ± 0.6	4.8 ± 0.6	< 0.001
HGB, g/L	137.6 ± 19.6	130.3 ± 19.6	135.7 ± 19.4	139.9 ± 17.9	144.2 ± 18.8	< 0.001
PLT, ×10^9^/L	241.9 ± 64.3	234.3 ± 65.6	242.0 ± 62.8	244.3 ± 65.0	247.0 ± 63.3	< 0.001
ALB, g/L	42.7 ± 4.0	41.9 ± 4.1	42.5 ± 4.0	42.9 ± 3.9	43.3 ± 3.8	< 0.001
AST, U/L	22.0 (18.0, 27.0)	21.0 (17.0, 25.0)	21.0 (18.0, 26.0)	22.0 (18.0, 27.0)	24.0 (20.0, 31.0)	< 0.001
ALT, U/L	20.0 (14.0, 29.0)	15.0 (12.0, 21.0)	18.0 (14.0, 26.0)	21.0 (15.0, 30.0)	26.0 (18.0, 38.0)	< 0.001
GGT, U/L	23.0 (15.0, 38.0)	15.0 (12.0, 22.0)	20.0 (15.0, 30.0)	25.0 (17.0, 38.5)	37.0 (23.0, 63.0)	< 0.001
TG, mmol/L	1.3 (0.9, 1.9)	0.7 (0.6, 0.8)	1.1 (0.9, 1.2)	1.6 (1.3, 1.8)	2.7 (2.2, 3.5)	< 0.001
TC, mmol/L	5.3 ± 1.3	4.8 ± 1.1	5.2 ± 1.2	5.4 ± 1.2	5.7 ± 1.6	< 0.001
LDL-C, mmol/L	3.4 ± 0.9	3.0 ± 0.8	3.4 ± 0.9	3.6 ± 0.9	3.6 ± 1.0	< 0.001
HDL-C, mmol/L	1.3 ± 0.4	1.4 ± 0.4	1.3 ± 0.4	1.3 ± 0.3	1.2 ± 0.3	< 0.001
FBG, mmol/L	5.6 ± 1.7	4.8 ± 0.6	5.3 ± 0.8	5.7 ± 1.2	6.7 ± 2.5	< 0.001
BUN, mmol/L	5.2 ± 1.7	5.0 ± 1.8	5.1 ± 1.5	5.3 ± 1.6	5.6 ± 1.9	< 0.001
CREA, umol/L	69.7 ± 22.6	65.6 ± 26.5	67.5 ± 18.7	71.0 ± 19.7	74.6 ± 23.5	< 0.001
UA, umol/L	363.0 ± 99.1	321.5 ± 86.6	346.4 ± 90.1	375.9 ± 94.6	406.8 ± 103.0	< 0.001
CEA, ng/ml	1.8 (1.2, 2.8)	1.7 (1.1, 2.5)	1.8 (1.1, 2.7)	1.9 (1.2, 2.9)	2.0 (1.4, 3.1)	< 0.001
CA199, U/ml	8.1 (4.7, 14.0)	7.6 (4.3, 13.4)	7.6 (4.7, 12.7)	8.3 (4.6, 14.0)	9.2 (5.1, 15.1)	< 0.001
Colorectal adenomatous polyps, n (%)						< 0.001
Yes	2844 (66.7)	765 (72.3)	684 (65.3)	705 (65.7)	690 (63.7)	
No	1419 (33.3)	293 (27.7)	364 (34.7)	368 (34.3)	394 (36.3)	

Data are presented as count (percent), median (interquartile range [IQR]) or mean ± Standard Deviation (SD).

BMI, body mass index; SBP, systolic blood pressure; DBP, diastolic blood pressure; HBP, hypertension; CHD, coronary heart disease; WBC, white blood cell count; NEU, Neutrophils count; RBC, red blood cell count; HGB, hemoglobin concentration; PLT, platelet count; ALB, albumin; AST, Aspartate aminotransferase; ALT, Alanine aminotransferase; GGT, gamma-glutamyl transpeptidase; TG, triglycerides; TC, total cholesterol; LDL-C, low-density lipoprotein cholesterol; HDL-C, high-density lipoprotein cholesterol; FBG, fasting blood glucose; BUN, blood urea nitrogen; CREA, creatinine; UA, uric acid; CEA, carcinoembryonic antigen; CA199, carbohydrate antigen 199; TyG, triglyceride glucose index.

### Baseline characteristics by colorectal polyps status

**Table 2** compares characteristics between participants with (n=2844) and without (n=1419) colorectal polyps. The polyp group had significantly higher proportions of males, married individuals, smokers, drinkers, hypertension, diabetes, and higher TyG index quartile representation (all p ≤ 0.001). They also exhibited older age, higher BMI, elevated blood pressure, adverse metabolic profiles (higher TG, LDL-C), increased liver enzymes, renal markers, CEA, and most hematological indices (RBC, HGB; lower PLT, NEU) (all p ≤ 0.001), except for comparable WBC (p=0.096) and CA199 (p=0.464).

**Table 2:** The baseline characteristics between the colorectal adenomatous polyps group and the non-colorectal adenomatous polyps group.

Characteristic	Total (n = 4263)	Non-colorectal adenomatous polyps (n = 1419)	Colorectal adenomatous polyps (n = 2844)	*p*-value
Gender, n (%)				< 0.001
Male	2397 (56.2)	1468 (51.6)	929 (65.5)	
Female	1866 (43.8)	1376 (48.4)	490 (34.5)	
Age, years	53.9 ± 12.5	52.0 ± 12.9	57.5 ± 10.9	< 0.001
Marital, n (%)				< 0.001
Married	4097 (96.1)	2699 (94.9)	1398 (98.5)	
Single	166 (3.9)	145 (5.1)	21 (1.5)	
BMI, kg/m^2^	23.5 ± 3.5	23.3 ± 3.6	23.8 ± 3.5	< 0.001
SBP, mmHg	127.0 ± 17.8	125.7 ± 17.9	129.6 ± 17.3	< 0.001
DBP, mmHg	81.3 ± 11.4	80.8 ± 11.5	82.3 ± 11.1	< 0.001
Smoking, n (%)				< 0.001
Smoker	865 (20.3)	508 (17.9)	357 (25.2)	
Non-smoker	3398 (79.7)	2336 (82.1)	1062 (74.8)	
Drinking, n (%)				< 0.001
Drinker	694 (16.3)	416 (14.6)	278 (19.6)	
Non-drinker	3569 (83.7)	2428 (85.4)	1141 (80.4)	
Hypertension, n (%)				< 0.001
Yes	1022 (24.0)	606 (21.3)	416 (29.3)	
No	3241 (76.0)	2238 (78.7)	1003 (70.7)	
Diabetes, n (%)				0.025
Yes	464 (10.9)	288 (10.1)	176 (12.4)	
No	3799 (89.1)	2556 (89.9)	1243 (87.6)	
CHD, n (%)				0.027
Yes	130 (3.0)	75 (2.6)	55 (3.9)	
No	4133 (97.0)	2769 (97.4)	1364 (96.1)	
WBC, ×10^9^/L	6.3 ± 2.1	6.4 ± 2.2	6.3 ± 1.8	0.096
NEU, ×10^9^/L	3.8 ± 1.8	3.9 ± 2.0	3.7 ± 1.4	< 0.001
RBC, ×10^9^/L	4.6 ± 0.6	4.6 ± 0.6	4.7 ± 0.6	< 0.001
HGB, g/L	137.6 ± 19.6	135.8 ± 20.6	141.2 ± 16.9	< 0.001
PLT, ×10^9^/L	241.9 ± 64.3	244.4 ± 67.1	237.0 ± 58.0	< 0.001
ALB, g/L	42.7 ± 4.0	42.6 ± 4.2	42.8 ± 3.5	0.046
AST, U/L	22.0 (18.0, 27.0)	22.0 (18.0, 27.0)	22.0 (19.0, 28.0)	< 0.001
ALT, U/L	20.0 (14.0, 29.0)	19.0 (13.2, 29.0)	21.0 (15.0, 29.0)	< 0.001
GGT, U/L	23.0 (15.0, 38.0)	22.0 (15.0, 37.0)	24.0 (16.0, 40.0)	< 0.001
TG, mmol/L	1.3 (0.9, 1.9)	1.2 (0.9, 1.9)	1.3 (0.9, 2.0)	< 0.001
TC, mmol/L	5.3 ± 1.3	5.3 ± 1.4	5.3 ± 1.3	0.059
LDL-C, mmol/L	3.4 ± 0.9	3.4 ± 0.9	3.4 ± 0.9	0.024
HDL-C, mmol/L	1.3 ± 0.4	1.3 ± 0.4	1.3 ± 0.3	0.083
FBG, mmol/L	5.6 ± 1.7	5.6 ± 1.7	5.6 ± 1.5	0.906
BUN, mmol/L	5.2 ± 1.7	5.1 ± 1.7	5.5 ± 1.7	< 0.001
CREA, umol/L	69.7 ± 22.6	68.7 ± 22.1	71.9 ± 23.4	< 0.001
UA, umol/L	363.0 ± 99.1	357.1 ± 99.0	374.7 ± 98.4	< 0.001
CEA, ng/ml	1.8 (1.2, 2.8)	1.8 (1.2, 2.7)	2.0 (1.3, 3.0)	< 0.001
CA199, U/ml	8.1 (4.7, 14.0)	8.1 (4.8, 14.2)	7.9 (4.6, 13.5)	0.464
TyG	8.7 ± 0.7	8.7 ± 0.7	8.7 ± 0.7	< 0.001
TyG group, n (%)				< 0.001
Q1	1058 (24.8)	765 (26.9)	293 (20.6)	
Q2	1048 (24.6)	684 (24.1)	364 (25.7)	
Q3	1073 (25.2)	705 (24.8)	368 (25.9)	
Q4	1084 (25.4)	690 (24.3)	394 (27.8)	

Data are presented as count (percent), median (interquartile range [IQR]) or mean ± Standard Deviation (SD).

BMI, body mass index; SBP, systolic blood pressure; DBP, diastolic blood pressure; HBP, hypertension; CHD, coronary heart disease; WBC, white blood cell count; NEU, Neutrophils count; RBC, red blood cell count; HGB, hemoglobin concentration; PLT, platelet count; ALB, albumin; AST, Aspartate aminotransferase; ALT, Alanine aminotransferase; GGT, gamma-glutamyl transpeptidase; TG, triglycerides; TC, total cholesterol; LDL-C, low-density lipoprotein cholesterol; HDL-C, high-density lipoprotein cholesterol; FBG, fasting blood glucose; BUN, blood urea nitrogen; CREA, creatinine; UA, uric acid; CEA, carcinoembryonic antigen; CA199, carbohydrate antigen 199; TyG, triglyceride glucose index.

### Associations of TyG index and colorectal polyps

**Supplementary Table 3** and **Table 3** respectively presents the univariate and multivariable logistic regression analysis for the association of TyG index with colorectal adenomatous polyp prevalence. Per unit increase in TyG was significantly associated with elevated polyp risk in all models (Model 1 OR: 1.51, 95% CI: 1.37–1.67; Model 2 OR: 1.29, 95% CI: 1.17–1.43; Model 3 OR: 1.13, 95% CI: 1.01–1.27; all p-trend ≤ 0.05). When analyzed by quartiles, higher TyG quartiles demonstrated progressively increased ORs for adenomatous polyp prevalence compared to Q1 (reference) in all models. This positive trend remained statistically significant after full adjustment (Model 3 p-trend = 0.032). Specifically, in the fully adjusted Model 3, participants in Q3 (OR: 1.32, 95% CI: 1.07–1.63, p=0.009) and Q4 (OR: 1.26, 95% CI: 1.01–1.58, p=0.042) maintained significantly elevated odds. Restricted cubic spline analysis did not provide evidence of a nonlinear association between the TyG index and colorectal adenomatous polyp risk (P for nonlinearity = 0.459, **Supplementary Figure 2**), indicating that the association could be reasonably modeled as linear within the observed range. Overall, higher TyG index values were associated with higher odds of colorectal adenomatous polyps.

**Table 3:** Multivariable logistic regression analysis of TyG index and prevalence of colorectal adenomatous polyps.

Variables	Model 1		Model 2		Model 3	
	OR (95% CI)	*P* value	OR (95% CI)	*P* value	OR (95% CI)	*P* value
TyG						
Per unit increase	1.51 (1.37~1.67)	<0.001	1.29 (1.17~1.43)	<0.001	1.13 (1.01~1.27)	0.038
Quartiles						
Q1	1 (Ref.)		1 (Ref.)		1 (Ref.)	
Q2	1.56 (1.31~1.87)	<0.001	1.33 (1.11~1.60)	0.002	1.21 (1.00~1.47)	0.054
Q3	1.98 (1.65~2.37)	<0.001	1.57 (1.30~1.90)	<0.001	1.32 (1.07~1.63)	0.009
Q4	2.12 (1.76~2.54)	<0.001	1.61 (1.33~1.95)	<0.001	1.26 (1.01~1.58)	0.042
* P* for trend		<0.001		<0.001		0.032

Model 1: Unadjusted.

Model 2: Adjusted for age and gender.

Model 3: Adjusted for age, gender, BMI, smoking, drinking, hypertension, diabetes, CHD, NEU, LDL-C, and HDL-C.

OR, Odds Ratio; CI, Confidence Interval; BMI, body mass index; CHD, coronary heart disease; NEU, Neutrophils count; LDL-C, low-density lipoprotein cholesterol; HDL-C, high-density lipoprotein cholesterol.

### Sensitivity analysis

**Figure 2:**
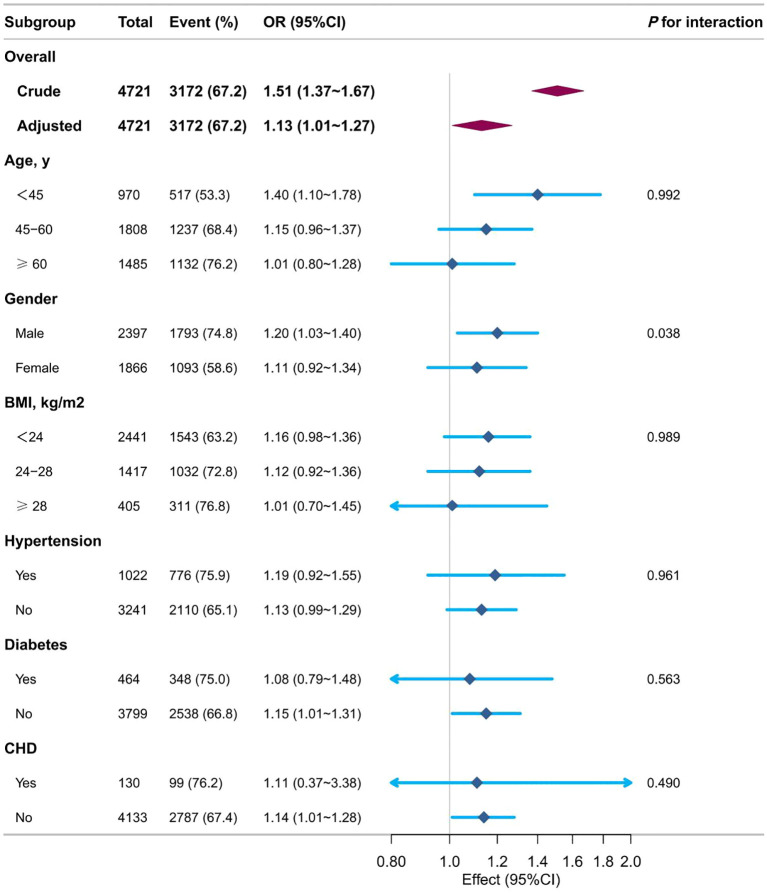
TyG index and colorectal adenomatous polyps subgroup analysis. Adjusted for age, gender, BMI, smoking, drinking, hypertension, diabetes, CHD, NEU, LDL-C, and HDL-C. OR, Odds Ratio; CI, Confidence Interval; BMI, body mass index; CHD, coronary heart disease; NEU, Neutrophils count; LDL-C, low-density lipoprotein cholesterol; HDL-C, high-density lipoprotein cholesterol; TyG, triglyceride glucose index.

Subgroup analyses were conducted stratifying by age, sex, BMI, and comorbidity status (**Figure 2**). The forest plot visually represents the consistency of the positive association between the TyG index and colorectal adenomatous polyps across all subgroups. Formal interaction tests revealed no significant effect modification by these factors, further strengthening the robustness of the primary findings. Furthermore, following multiple imputation for missing covariates, multivariable logistic regression analyses were repeated using the imputed datasets (**Supplementary Table 4**). The results indicated a persistent positive association between the TyG index and the prevalence of colorectal adenomatous polyps. Notably, a statistically significant dose-response relationship was observed across TyG quartiles in the fully adjusted model (Model 3), with a P-for-trend of 0.032. Although the odds ratios (ORs) were attenuated after comprehensive adjustment for confounders including age, gender, BMI, lifestyle factors, comorbidities, and laboratory parameters, the graded association remained evident. The sensitivity analyses, encompassing both extensive subgroup analysis and multiple imputation, confirm the stable and independent association of the TyG index with an increased prevalence of colorectal adenomatous polyps.

### Genetic associations with TyG index and colorectal polyps

**Figure 3:**
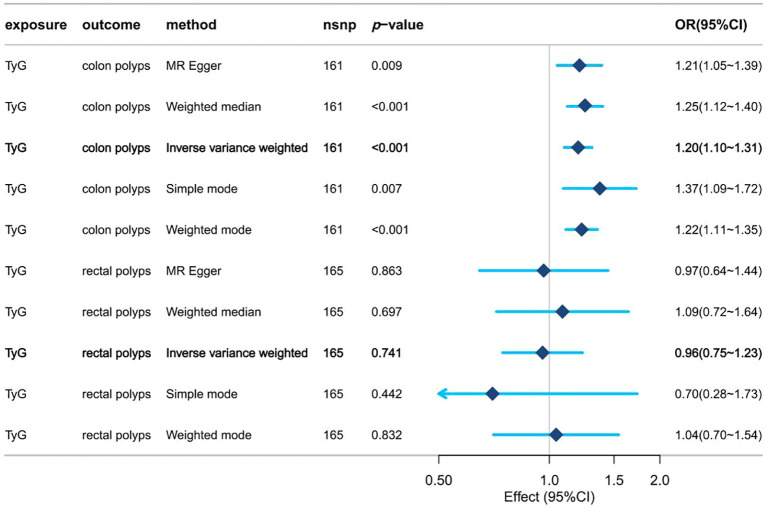
Mendelian randomization (MR) association between genetically determined TyG index and colorectal polyps. OR, Odds Ratio; CI, Confidence Interval; TyG, triglyceride glucose index; nsnp, number of SNP.

**Figure 4:**
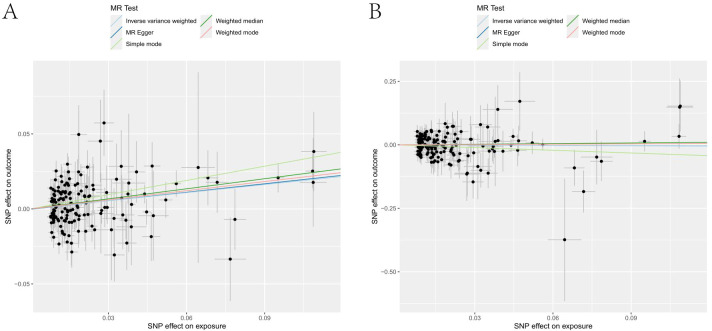
The scatter plot of genetic association of TyG index with **(A)** colon polyps and **(B)** rectal polyps.

Genetically predicted TyG index elevation demonstrated a significant association with increased colon polyps risk (IVW OR: 1.20; 95% CI: 1.10-1.31; p < 0.001). Sensitivity analyses using MR-Egger and three other MR methods yielded consistent directional estimates (**Figure 3**). In the scatter plot, the upward slope of regression lines (IVW/MR-Egger) visually corroborated the positive causal effect of genetically elevated TyG index on colon polyp risk (**Figure 4**). However, Mendelian randomization analysis revealed no significant association between genetically instrumented TyG index and rectal polyps risk (IVW OR: 0.96; 95% CI: 0.75-1.23; p = 0.741) (**Figure 3**). In **Figure 4**, consistently flat slopes across methods aligned with the null MR association. Sensitivity analyses confirmed the null association (**Figure 3**). No evidence of directional pleiotropy was detected for colon polyps (intercept: −0.00021, SE = 0.001, p=0.884) or rectal polyps (intercept: −0.00017, SE = 0.004, p=0.967) (**Table 4**). Significant heterogeneity existed for colon polyps (IVW Q = 280.78, df=160, p<0.001; MR-Egger Q = 280.74, df=159, p<0.001) and no significant heterogeneity was observed for rectal polyps (**Supplementary Table 5**, **Supplementary Figure 3**).

**Table 4:** Pleiotropy assessment of genetic association of TyG index with colorectal polyps.

Exposure	Outcome	Egger_intercept	SE	Pval
TyG	colon polyps	-0.000210305	0.001	0.884
TyG	rectal polyps	-0.000176007	0.004	0.967

## Discussion

This large-scale cross-sectional study of 4,263 participants, combined with a two-sample Mendelian randomization analysis based on GWAS datasets, demonstrated that a higher TyG index is an independent risk factor for colorectal polyps. After comprehensive adjustment for covariates, each one-unit increase in the TyG index was associated with 13% higher odds of colorectal adenomatous polyps. Specifically, the study revealed a causal association between elevated TyG index and colon polyps, but not rectal polyps. These results remained consistent following sensitivity analyses. The findings further suggest that insulin resistance may play a pathogenic role in the development of colon polyps, underscoring the importance of considering the marker of insulin resistance, TyG index, when assessing an individual’s risk for colon neoplasia.

Our findings align with a growing body of evidence implicating insulin resistance (IR) as a significant risk factor in the early pathogenesis of colorectal neoplasia. Multiple epidemiological studies have consistently reported associations between IR, often assessed by hyperinsulinemia or the homeostasis model assessment (HOMA-IR), and an increased prevalence of colorectal adenomas, a known precursor to colorectal cancer (CRC) (25–28). A meta-analysis by Yoon et al. solidified this link, demonstrating that both hyperinsulinemia and IR were significantly associated with an elevated risk of colorectal adenomas (27). However, the clinical utility of HOMA-IR is often constrained by the need for insulin measurement, which is not routinely performed in all settings due to cost and accessibility. Our study advances this field by validating the TyG index, a robust and easily calculable surrogate marker of IR. The results from our cross-sectional analysis are consistent with the recent work of Xu et al., who also identified TyG, alongside TyG-BMI, TG/HDL-C, and METS-IR, as significant risk factors for colorectal polyps, with predictive performances surpassing that of the traditional tumor marker CEA (22). We extend these observational findings by providing crucial genetic evidence from our Mendelian randomization analysis, which supports a potential causal relationship between a genetically predisposed higher TyG index and increased odds of developing colon polyps. This MR approach significantly strengthens the inference by mitigating concerns of reverse causality and residual confounding inherent in observational designs. While previous research, such as a nationwide study by Luo et al., has explored the association between IR surrogates and CRC incidence (29), direct evidence specifically focusing on colorectal polyps, particularly from an MR perspective, has been scarce. Furthermore, the study by Li et al. highlights the complex interplay between inflammation, metabolism, and polyps, demonstrating that the TyG index mediates the association between a novel inflammatory-nutritional index (CALLY) and polyp prevalence, an effect particularly pronounced in women (30). This underscores the TyG index’s role within a broader metabolic-inflammatory network influencing colorectal carcinogenesis. Therefore, our study fills a critical gap by not only corroborating the observational association but also providing novel genetic evidence that bolsters the case for a causal role of insulin resistance, as captured by the TyG index, in the development of colon polyps.

The biological plausibility of our findings is strongly supported by several interconnected pathways through which insulin resistance (IR) likely promotes colorectal polyp development. The most prominent mechanism involves the state of chronic hyperinsulinemia that characterizes compensated IR. Elevated insulin levels exert direct mitogenic and anti-apoptotic effects on colonic epithelial cells by binding to insulin receptors and, with lower affinity, to insulin-like growth factor-1 receptors (IGF-1R). This activates downstream oncogenic signaling cascades, notably the MAPK and PI3K/Akt pathways, which drive cellular proliferation and inhibit programmed cell death (31, 32).

Concurrently, IR is frequently associated with alterations in the IGF-1 axis. This includes increased bioavailable IGF-1, a potent mitogen, and reduced levels of its binding protein, IGFBP-3. The resulting imbalance creates a microenvironment highly conducive to the uncontrolled growth and survival of initiated epithelial cells, facilitating the formation and progression of adenomatous polyps (33, 34). Furthermore, IR is intrinsically linked to a state of chronic, low-grade inflammation. This pro-inflammatory milieu, characterized by elevated levels of cytokines such as TNF-α and IL-6, not only directly damages DNA and promotes genomic instability but also further exacerbates peripheral IR, creating a vicious cycle that fuels tumorigenesis (35).

The components of the TyG index itself—triglycerides and glucose—also contribute directly to the pathogenic landscape. Dyslipidemia, particularly high triglycerides, can promote oxidative stress and membrane instability (36). Hyperglycemia facilitates the formation of advanced glycation end-products (AGEs), which have been implicated in cellular dysfunction and proliferation (37). Moreover, emerging evidence highlights the role of local “lipotoxicity” within the colonic tissue. Under conditions of IR, increased supply of free fatty acids to the colorectal mucosa leads to ectopic triglyceride accumulation and lipid droplet formation within the submucosa, a phenomenon directly associated with abdominal obesity and IR (38). This local fat deposition is believed to act as a paracrine source of pro-inflammatory adipokines, further enriching the pro-tumorigenic niche (39). Thus, the TyG index effectively encapsulates a glucolipotoxic environment that drives both systemic metabolic dysfunction and localized cellular changes, collectively fostering the development of colon polyps.

The site-specific genetic association observed in our study may reflect biological differences between colon and rectal tumorigenesis. Compared with the rectum, the colon is more extensively exposed to bile acid metabolism, microbiota-derived metabolites, and systemic metabolic disturbances (40) (41). Insulin resistance may promote colonic epithelial proliferation through hyperinsulinemia, IGF-1 signaling, oxidative stress, chronic low-grade inflammation, and glucolipotoxicity (42). In contrast, rectal lesions may be influenced to a greater extent by local anatomical, mechanical, inflammatory, or environmental factors. Moreover, colon and rectal lesions differ in embryological origin and molecular carcinogenic profiles, which may contribute to heterogeneous associations with TyG index (40–43). Although the MR analysis allowed separate evaluation of colon and rectal polyps using site-specific GWAS outcomes, corresponding site-specific observational analyses were not performed because the clinical cohort was designed to assess patient-level colorectal adenomatous polyp prevalence rather than lesion-level anatomical outcomes. In particular, synchronous adenomas at multiple colorectal sites may lead to overlapping outcome categories, and *post hoc* anatomical stratification could introduce misclassification and unstable estimates.

Significant heterogeneity was observed in the MR analysis for colon polyps. This may reflect the complex polygenic architecture of the TyG index and the possibility that individual genetic variants influence metabolic pathways through partially different biological mechanisms. Nevertheless, the direction of effect was generally consistent across multiple MR methods, and MR-Egger intercept analysis did not indicate significant directional pleiotropy. Therefore, while the heterogeneity suggests that the MR findings should be interpreted cautiously, it does not necessarily invalidate the observed association between genetically predicted TyG index and colon polyps.

The primary strength of this study lies in the complementary application of a large-scale cross-sectional analysis and a Mendelian randomization approach. The consistency of the positive association between the TyG index and colon polyp risk across both methodologies substantially strengthens the inference of a potential causal relationship. The MR design, by leveraging genetic variants fixed at conception, effectively minimizes concerns regarding reverse causality and unmeasured confounding that typically plague observational studies. Furthermore, the use of the TyG index, derived from routine and inexpensive laboratory measures (fasting triglycerides and glucose), underscores the high translational potential and clinical accessibility of our findings compared to studies reliant on insulin-based markers like HOMA-IR.

Notwithstanding these strengths, several limitations warrant careful consideration. Firstly, despite employing robust MR methods and sensitivity analyses to assess pleiotropy, we cannot entirely rule out the possibility that the genetic instruments influenced polyp risk through pathways independent of the TyG index. Secondly, our study population was recruited from a single health management center, which may limit the generalizability of our findings to other ethnic or demographic groups. Thirdly, we lacked detailed data on certain potential confounders, including dietary habits and physical activity patterns, which could residually confound the observed associations. Future studies incorporating these variables are essential. Fourthly, the GWAS dataset for rectal polyps included substantially fewer cases than that for colon polyps, which may have reduced the statistical power to detect modest causal effects. Therefore, the null MR result for rectal polyps should be interpreted cautiously and should not be regarded as conclusive evidence of absence of association. Future MR studies using larger rectal polyp GWAS datasets are warranted. Finally, another limitation is that our observational cohort did not permit robust lesion-level site-specific analyses for colon and rectal adenomatous polyps. Therefore, the site-specific findings were mainly derived from the MR analysis and should be further validated in future prospective cohorts with standardized lesion-level endoscopic and pathological data.

## Conclusions

In conclusion, this integrated study provides compelling evidence from both observational and genetic perspectives that insulin resistance, as accurately reflected by the TyG index, is significantly and likely causally associated with an increased prevalence of colorectal adenomatous polyps. The TyG index emerges as a simple, cost-effective, and robust biomarker that could be valuable for risk stratification, potentially helping to identify individuals who might benefit from earlier or more intensive colorectal cancer screening. These findings reinforce the importance of metabolic health in the early pathogenesis of colorectal neoplasia and pave the way for future research, including prospective cohort studies to confirm temporality and interventional trials to determine if improving insulin sensitivity can reduce polyp incidence.

## Data Availability

The clinical data analysed in this study are not publicly available due to ethical and privacy restrictions. Data may be made available from the corresponding author upon reasonable request and with approval from the relevant institutional review board. Publicly available GWAS summary statistics were used for the Mendelian randomization analysis.
