# Supplementary material for: TyG index is associated with colorectal adenomatous polyps and genetically linked to colon polyps: a cross-sectional and Mendelian randomization study
Source: Front Oncol. 2026 Jul 8;16:1756399. doi: 10.3389/fonc.2026.1756399 (PMC13388076; doi:10.3389/fonc.2026.1756399)

**Association between TyG index and risk of colorectal polyps: a cross-sectional and Mendelian randomization study**

Yingyi Li^1^**^†^**, Liangfu Guo^2^**^†^**, Xiaodong Zhu^1^, Chanchan Lin^1^, Xiaoqiang Liu^1^, Zicheng Huang^1^**^†^**, Yisen Huang^1^**^†^**^*^

**Supplementary Table S1** 192 SNPs significantly associated with TyG index (p<5x10^-8^ and r^2^<0.01 for TyG)

| SNP | other allele | effect allele | β | SE | *P*-value |
| --- | --- | --- | --- | --- | --- |
| rs114165349 | G | C | 0.0445298 | 0.00465259 | 1.07E-21 |
| rs72904790 | T | C | -0.0137147 | 0.00240632 | 1.20E-08 |
| rs213498 | T | A | -0.00802754 | 0.00146386 | 4.17E-08 |
| rs10889332 | C | T | -0.0388967 | 0.00143046 | 1.34E-162 |
| rs72669514 | C | T | 0.018609 | 0.00320686 | 6.53E-09 |
| rs17656269 | C | T | 0.00874937 | 0.00147002 | 2.65E-09 |
| rs16836630 | G | C | -0.0173377 | 0.00252122 | 6.14E-12 |
| rs1760801 | G | A | -0.00895698 | 0.0015125 | 3.19E-09 |
| rs340836 | T | C | -0.00869811 | 0.00139646 | 4.71E-10 |
| rs76172548 | A | C | 0.0228139 | 0.00383242 | 2.64E-09 |
| rs3120619 | G | A | 0.0118809 | 0.0018033 | 4.45E-11 |
| rs11118610 | A | C | -0.00902819 | 0.00138913 | 8.09E-11 |
| rs4846922 | C | T | 0.0221545 | 0.00146286 | 8.63E-52 |
| rs907866 | G | A | -0.00927654 | 0.00138756 | 2.31E-11 |
| rs111585158 | C | T | 0.0121133 | 0.00211397 | 1.00E-08 |
| rs144470864 | A | C | 0.0172612 | 0.00307793 | 2.05E-08 |
| rs76384951 | A | C | -0.0295509 | 0.0025237 | 1.16E-31 |
| rs533617 | T | C | -0.0464535 | 0.00346743 | 6.48E-41 |
| rs35750610 | T | C | 0.0185128 | 0.00234709 | 3.09E-15 |
| rs34921778 | A | G | 0.00842606 | 0.00144997 | 6.21E-09 |
| rs12617848 | C | T | 0.0142794 | 0.00200585 | 1.09E-12 |
| rs80216311 | C | T | -0.0141686 | 0.00238957 | 3.05E-09 |
| rs61737373 | G | A | -0.028607 | 0.00292878 | 1.57E-22 |
| rs6547692 | A | G | 0.0375073 | 0.00138448 | 2.05E-161 |
| rs10206462 | T | C | -0.00893744 | 0.00142769 | 3.85E-10 |
| rs6760053 | C | G | -0.00778921 | 0.00137713 | 1.55E-08 |
| rs6710938 | A | C | -0.00898973 | 0.00161797 | 2.76E-08 |
| rs79953491 | A | G | -0.0237048 | 0.00210804 | 2.49E-29 |
| rs115128825 | C | A | 0.026901 | 0.00487631 | 3.46E-08 |
| rs484066 | T | A | -0.0159198 | 0.00141892 | 3.31E-29 |
| rs17694506 | T | C | 0.00900471 | 0.00141859 | 2.19E-10 |
| rs2943645 | T | C | -0.0209209 | 0.0014316 | 2.40E-48 |
| rs6437249 | C | T | 0.00838469 | 0.00149284 | 1.95E-08 |
| rs147764624 | G | C | -0.03019 | 0.00550582 | 4.18E-08 |
| rs390802 | G | A | -0.0153354 | 0.00176462 | 3.63E-18 |
| rs62271373 | T | A | 0.0253586 | 0.00303878 | 7.16E-17 |
| rs13074711 | T | C | 0.0120107 | 0.00218488 | 3.86E-08 |
| rs13108218 | G | A | 0.0156395 | 0.00143815 | 1.54E-27 |
| rs71603401 | A | G | 0.0125089 | 0.00205483 | 1.15E-09 |
| rs6448429 | C | T | 0.0126753 | 0.00187808 | 1.49E-11 |
| rs1471251 | A | T | 0.0164471 | 0.00140761 | 1.56E-31 |
| rs4134363 | G | A | -0.00950005 | 0.00170166 | 2.37E-08 |
| rs3822076 | T | A | 0.00867089 | 0.0013818 | 3.50E-10 |
| rs2035816 | A | G | -0.0159447 | 0.00249077 | 1.54E-10 |
| rs78025076 | C | T | 0.0271124 | 0.00482777 | 1.96E-08 |
| rs390556 | T | C | -0.0132704 | 0.00220378 | 1.73E-09 |
| rs72754154 | G | A | -0.0213999 | 0.00314107 | 9.58E-12 |
| rs3936511 | A | G | 0.0216126 | 0.00174659 | 3.69E-35 |
| rs151913 | G | A | 0.00786984 | 0.00141384 | 2.60E-08 |
| rs7703744 | C | G | -0.0109345 | 0.00155172 | 1.84E-12 |
| rs72801474 | G | A | -0.0146422 | 0.00235286 | 4.88E-10 |
| rs12173130 | T | C | 0.00971075 | 0.00176945 | 4.07E-08 |
| rs11134475 | G | A | -0.0169417 | 0.00142319 | 1.15E-32 |
| rs2963476 | A | G | 0.0133691 | 0.00169886 | 3.58E-15 |
| rs6923241 | C | T | -0.0109234 | 0.00154721 | 1.67E-12 |
| rs2745400 | G | A | 0.00826032 | 0.00137239 | 1.76E-09 |
| rs2894211 | C | A | 0.0174652 | 0.00218851 | 1.46E-15 |
| rs7758790 | T | C | 0.0142803 | 0.00164795 | 4.52E-18 |
| rs55697600 | A | G | 0.0351072 | 0.00360432 | 2.05E-22 |
| rs185139895 | G | A | 0.0207564 | 0.00338431 | 8.63E-10 |
| rs3025053 | G | A | -0.0134581 | 0.00212481 | 2.40E-10 |
| rs4715317 | G | T | 0.00974329 | 0.00143947 | 1.30E-11 |
| rs1967685 | G | C | -0.0142769 | 0.0013714 | 2.25E-25 |
| rs632057 | G | T | 0.0153665 | 0.00142077 | 2.94E-27 |
| rs12208357 | C | T | 0.0219558 | 0.00272455 | 7.75E-16 |
| rs77009508 | A | G | 0.0224178 | 0.00259593 | 5.86E-18 |
| rs55730499 | C | T | -0.0182481 | 0.0025524 | 8.74E-13 |
| rs186696265 | C | T | -0.0472632 | 0.00592501 | 1.51E-15 |
| rs4709746 | C | T | -0.0112466 | 0.00203182 | 3.11E-08 |
| rs852424 | C | T | 0.00852818 | 0.0014628 | 5.55E-09 |
| rs38205 | C | A | 0.00791466 | 0.00143983 | 3.87E-08 |
| rs2106727 | G | A | -0.0108194 | 0.00142823 | 3.59E-14 |
| rs4722551 | T | C | -0.0185803 | 0.00188091 | 5.21E-23 |
| rs1534696 | A | C | 0.0107368 | 0.00137638 | 6.18E-15 |
| rs2971676 | G | A | 0.0133494 | 0.00239848 | 2.61E-08 |
| rs878521 | G | A | 0.0217619 | 0.00158662 | 8.43E-43 |
| rs62459110 | G | C | -0.0213924 | 0.00364332 | 4.32E-09 |
| rs799157 | C | T | 0.0407906 | 0.00340184 | 4.05E-33 |
| rs17145750 | C | T | -0.0560614 | 0.00185642 | 5.28E-200 |
| rs10260148 | C | T | 0.0149589 | 0.00154174 | 2.96E-22 |
| rs73198299 | T | C | 0.0122709 | 0.00222876 | 3.68E-08 |
| rs7821812 | G | C | 0.0163357 | 0.00169741 | 6.39E-22 |
| rs904009 | A | C | 0.0159306 | 0.00162585 | 1.16E-22 |
| rs4921914 | T | C | 0.0194386 | 0.00165914 | 1.07E-31 |
| rs2975424 | T | C | 0.0106046 | 0.0017554 | 1.53E-09 |
| rs1388941 | G | A | 0.0143536 | 0.00145941 | 8.01E-23 |
| rs268 | A | G | 0.10865 | 0.00515243 | 1.25E-98 |
| rs117026536 | G | T | -0.0951759 | 0.00226332 | 0 |
| rs57295072 | G | C | -0.0304863 | 0.00469748 | 8.60E-11 |
| rs17091881 | T | C | 0.0785949 | 0.00427426 | 1.82E-75 |
| rs74444445 | T | C | 0.034928 | 0.00488632 | 8.82E-13 |
| rs117805502 | C | T | -0.0321654 | 0.00439412 | 2.48E-13 |
| rs28550053 | A | G | -0.0177064 | 0.00182104 | 2.42E-22 |
| rs75662196 | G | C | -0.0279294 | 0.00434519 | 1.30E-10 |
| rs17092008 | C | T | 0.0208253 | 0.00285324 | 2.91E-13 |
| rs11781356 | T | A | 0.00993171 | 0.00176553 | 1.85E-08 |
| rs2081687 | C | T | 0.011677 | 0.00145382 | 9.63E-16 |
| rs71525127 | C | G | 0.019603 | 0.00254745 | 1.42E-14 |
| rs11558471 | A | G | -0.011475 | 0.00147145 | 6.29E-15 |
| rs17321515 | A | G | -0.0439 | 0.00137141 | 2.01E-224 |
| rs62521590 | T | G | 0.0146537 | 0.00155763 | 5.11E-21 |
| rs10811661 | T | C | -0.00987338 | 0.0018054 | 4.53E-08 |
| rs13289566 | C | T | -0.0118284 | 0.00166888 | 1.37E-12 |
| rs2244278 | C | A | -0.0133902 | 0.00211876 | 2.62E-10 |
| rs3750571 | C | A | -0.0124087 | 0.00189812 | 6.27E-11 |
| rs11006681 | G | A | -0.0110061 | 0.00184368 | 2.38E-09 |
| rs142164605 | T | A | -0.0177614 | 0.00278129 | 1.71E-10 |
| rs10786069 | T | C | 0.0130977 | 0.00137832 | 2.06E-21 |
| rs113344423 | G | A | 0.0212993 | 0.00301795 | 1.70E-12 |
| rs2792736 | A | T | -0.0100505 | 0.00153811 | 6.40E-11 |
| rs10832027 | A | G | -0.0122569 | 0.00148245 | 1.37E-16 |
| rs3808976 | A | G | 0.00981886 | 0.00170108 | 7.84E-09 |
| rs99780 | C | T | 0.020203 | 0.00143553 | 5.73E-45 |
| rs35169799 | C | T | 0.0247241 | 0.00283283 | 2.61E-18 |
| rs678614 | C | A | 0.00935072 | 0.00153217 | 1.04E-09 |
| rs2302883 | T | C | 0.00886478 | 0.00162296 | 4.71E-08 |
| rs187217942 | G | A | 0.031159 | 0.00540272 | 8.06E-09 |
| rs17119701 | A | G | 0.0370675 | 0.00375036 | 4.94E-23 |
| rs61362984 | A | G | -0.0139461 | 0.00142207 | 1.06E-22 |
| rs61904855 | C | A | 0.0233784 | 0.00409645 | 1.15E-08 |
| rs11216122 | G | T | -0.0181908 | 0.00310342 | 4.59E-09 |
| rs7930786 | G | C | 0.124688 | 0.00278666 | 0 |
| rs56225305 | G | A | 0.108415 | 0.00278601 | 0 |
| rs2075294 | G | T | 0.0388118 | 0.00579905 | 2.19E-11 |
| rs75919952 | C | T | -0.046805 | 0.0031699 | 2.56E-49 |
| rs11600380 | T | C | -0.03673 | 0.00254055 | 2.34E-47 |
| rs5110 | C | A | -0.0185398 | 0.00248476 | 8.59E-14 |
| rs12721078 | C | A | -0.0322797 | 0.0039376 | 2.46E-16 |
| rs71480323 | G | A | -0.0195603 | 0.00211444 | 2.24E-20 |
| rs11216236 | C | T | 0.0240392 | 0.00342403 | 2.21E-12 |
| rs187929675 | C | T | -0.0767868 | 0.00606752 | 1.07E-36 |
| rs11045171 | A | G | -0.0116623 | 0.00173721 | 1.91E-11 |
| rs67981690 | A | G | 0.0148562 | 0.00207488 | 8.09E-13 |
| rs10783828 | G | A | 0.00903509 | 0.00147493 | 9.04E-10 |
| rs7296326 | T | C | -0.0118561 | 0.00217251 | 4.84E-08 |
| rs1585705 | A | C | 0.00876614 | 0.001496 | 4.64E-09 |
| rs10861679 | T | C | 0.00935439 | 0.00150663 | 5.35E-10 |
| rs1882491 | T | C | -0.0134975 | 0.00148108 | 8.04E-20 |
| rs1716407 | A | G | -0.0150613 | 0.00139861 | 4.90E-27 |
| rs7140110 | T | C | 0.0143695 | 0.00150803 | 1.61E-21 |
| rs112740904 | T | G | -0.0149023 | 0.00195835 | 2.76E-14 |
| rs12885801 | C | A | 0.00908515 | 0.00162151 | 2.11E-08 |
| rs34820917 | G | A | -0.0157999 | 0.00285011 | 2.97E-08 |
| rs35477346 | T | C | 0.00929684 | 0.00149721 | 5.32E-10 |
| rs139974673 | T | C | 0.0717689 | 0.00443008 | 5.34E-59 |
| rs72739147 | A | T | -0.0121232 | 0.00206079 | 4.04E-09 |
| rs1532085 | G | A | 0.0180035 | 0.0014104 | 2.64E-37 |
| rs261334 | C | G | 0.0261448 | 0.00167767 | 9.88E-55 |
| rs11636087 | T | C | 0.0116533 | 0.00154515 | 4.65E-14 |
| rs8028620 | T | C | -0.00897866 | 0.00137468 | 6.53E-11 |
| rs7175132 | A | G | -0.00811532 | 0.00141367 | 9.44E-09 |
| rs8025505 | C | T | 0.00964703 | 0.00158419 | 1.13E-09 |
| rs9935836 | A | C | 0.00988172 | 0.00177279 | 2.49E-08 |
| rs11075253 | C | A | -0.0141141 | 0.00150196 | 5.65E-21 |
| rs12446515 | C | T | -0.0187602 | 0.00147498 | 4.76E-37 |
| rs5880 | G | C | 0.0221143 | 0.00298074 | 1.18E-13 |
| rs12934528 | T | C | 0.0135148 | 0.00195818 | 5.15E-12 |
| rs2925979 | C | T | 0.0154375 | 0.00149811 | 6.79E-25 |
| rs11651957 | G | A | 0.0186479 | 0.00293615 | 2.14E-10 |
| rs12937081 | A | G | 0.0108836 | 0.00189096 | 8.64E-09 |
| rs72836561 | C | T | 0.0682178 | 0.00389919 | 1.69E-68 |
| rs231539 | C | T | 0.0130645 | 0.00188077 | 3.76E-12 |
| rs11657238 | G | A | -0.00784868 | 0.00138378 | 1.41E-08 |
| rs1801689 | A | C | -0.0293183 | 0.00408097 | 6.78E-13 |
| rs77244849 | T | C | -0.00875261 | 0.00148835 | 4.09E-09 |
| rs9891030 | G | A | 0.00993826 | 0.00159406 | 4.54E-10 |
| rs71352934 | A | C | -0.0163114 | 0.00273609 | 2.50E-09 |
| rs8092347 | A | G | 0.00812063 | 0.00141115 | 8.69E-09 |
| rs197156 | A | G | -0.00925016 | 0.00145381 | 1.99E-10 |
| rs1035941 | G | A | 0.0110148 | 0.00153309 | 6.75E-13 |
| rs4804413 | C | T | 0.00948499 | 0.00138516 | 7.53E-12 |
| rs116843064 | G | A | -0.108878 | 0.00491157 | 8.79E-109 |
| rs57192995 | G | C | -0.019903 | 0.00302387 | 4.65E-11 |
| rs58542926 | C | T | -0.0520456 | 0.00257716 | 1.26E-90 |
| rs188247550 | C | T | -0.0644712 | 0.00642107 | 1.02E-23 |
| rs62102718 | A | T | 0.0115887 | 0.00152691 | 3.22E-14 |
| rs58895965 | C | A | 0.0127238 | 0.00180395 | 1.75E-12 |
| rs541012177 | G | T | 0.0242543 | 0.00353606 | 6.94E-12 |
| rs41290102 | C | T | -0.0331177 | 0.00588779 | 1.86E-08 |
| rs419925 | G | C | -0.0130424 | 0.00149941 | 3.39E-18 |
| rs483082 | G | T | 0.0446753 | 0.00161686 | 8.09E-168 |
| rs79429216 | G | A | 0.0378212 | 0.0062985 | 1.92E-09 |
| rs146390218 | A | G | 0.0355235 | 0.00435052 | 3.22E-16 |
| rs62132802 | C | T | -0.00911832 | 0.00150245 | 1.29E-09 |
| rs12610709 | G | A | 0.013994 | 0.00183258 | 2.24E-14 |
| rs2207132 | G | A | 0.0283225 | 0.00387832 | 2.83E-13 |
| rs2250900 | C | T | 0.00899617 | 0.00163964 | 4.10E-08 |
| rs6073958 | T | C | 0.0274094 | 0.00172428 | 7.14E-57 |
| rs4812995 | T | C | 0.0091331 | 0.00161739 | 1.64E-08 |
| rs6066138 | G | A | -0.00850564 | 0.00152344 | 2.36E-08 |
| rs6090040 | C | A | 0.0089174 | 0.00138822 | 1.33E-10 |
| rs2277844 | A | G | -0.00908433 | 0.00138503 | 5.43E-11 |

**Supplementary Table S2** Summary of the GWAS included in this study.

| Variables | ID | Population | Sample size | Case size | Sex | Year |
| --- | --- | --- | --- | --- | --- | --- |
| TyG index | UKB | European | 273368 | - | Males and Females | 2024 |
| colon polyps | GCST90475319 | European | 315668 | 73737 | Males and Females | 2024 |
| rectal polyps | GCST90478441 | European | 435846 | 3409 | Males and Females | 2024 |

**Supplementary Table S3** Univariate logistic regression analysis of factors influencing colorectal polyps occurrence.

| **Characteristic** | **OR (95% CI)** | ***p*-value** |
| --- | --- | --- |
| Female | 0.47 (0.42~0.54) | <0.001 |
| Age | 1.03 (1.03~1.04) | <0.001 |
| Married | 2.72 (2.02~3.68) | <0.001 |
| BMI | 1.10 (1.08~1.12) | <0.001 |
| SBP | 1.01 (1.01~1.02) | <0.001 |
| DBP | 1.02 (1.01~1.02) | <0.001 |
| Smoker | 2.06 (1.74~2.44) | <0.001 |
| Drinker | 2.00 (1.66~2.41) | <0.001 |
| Hypertension | 1.62 (1.4~1.88) | <0.001 |
| Diabetes | 1.32 (1.08~1.61) | 0.006 |
| CHD | 1.48 (1.02~2.15) | 0.038 |
| WBC | 0.99 (0.97~1.02) | 0.663 |
| NEU | 0.96 (0.93~0.99) | 0.007 |
| RBC | 1.55 (1.41~1.71) | <0.001 |
| HGB | 1.02 (1.02~1.03) | <0.001 |
| PLT | 1.00 (1.00~1.00) | <0.001 |
| ALB | 1.06 (1.05~1.08) | <0.001 |
| AST | 1.00 (1.00~1.00) | 0.597 |
| ALT | 1.00 (1.00~1.00) | 0.025 |
| GGT | 1.00 (1.00~1.00) | 0.039 |
| TG | 1.11 (1.06~1.16) | <0.001 |
| TC | 1.14 (1.08~1.19) | <0.001 |
| LDL-C | 1.21 (1.14~1.30) | <0.001 |
| HDL-C | 0.83 (0.70~0.98) | 0.025 |
| FBG | 1.06 (1.02~1.10) | 0.005 |
| BUN | 1.08 (1.04~1.12) | <0.001 |
| CREA | 1.01 (1.00~1.01) | <0.001 |
| UA | 1.00 (1.00~1.00) | <0.001 |
| CEA | 1.08 (1.04~1.12) | <0.001 |
| CA199 | 1.00 (1.00~1.00) | 0.404 |
| TyG | 1.47 (1.34~1.61) | <0.001 |
| Q1 | 1 (Ref.) |  |
| Q2 | 1.53 (1.30~1.81) | <0.001 |
| Q3 | 1.96 (1.65~2.33) | <0.001 |
| Q4 | 1.99 (1.68~2.36) | <0.001 |

OR, Odds Ratio; CI, Confidence Interval; BMI, body mass index; SBP, systolic blood pressure; DBP, diastolic blood pressure; HBP, hypertension; CHD, coronary heart disease; WBC, white blood cell count; NEU, Neutrophils count; RBC, red blood cell count; HGB, hemoglobin concentration; PLT, platelet count; ALB, albumin; AST, Asparate aminotransferase; ALT, Alanine aminotransferase; GGT, gamma-glutamyl transpeptidase; TG, triglycerides; TC, total cholesterol; LDL-C, low-density lipoprotein cholesterol; HDL-C, high-density lipoprotein cholesterol; FBG, fasting blood glucose; BUN, blood urea nitrogen; CREA, creatinine; UA, uric acid; CEA, carcinoembryonic antigen; CA199, carbohydrate antigen 199; TyG, triglyceride glucose index

**Supplementary Table S4** Multivariable logistic regression analysis of TyG index and prevalence of colorectal polyps after multiple imputation

| **Variables** | **Model 1** | | **Model 2** | | **Model 3** | |
| --- | --- | --- | --- | --- | --- | --- |
|  | **OR (95% CI)** | ***P* value** | **OR (95% CI)** | ***P* value** | **OR (95% CI)** | ***P* value** |
| TyG |  |  |  |  |  |  |
| Per SD increase | 1.47 (1.34~1.61) | <0.001 | 1.27 (1.16~1.40) | <0.001 | 1.11 (0.99~1.24) | 0.067 |
| Quartiles |  |  |  |  |  |  |
| Q1 | 1 (Ref.) |  | 1 (Ref.) |  | 1 (Ref.) |  |
| Q2 | 1.53 (1.30~1.81) | <0.001 | 1.32 (1.11~1.57) | 0.002 | 1.19 (0.99~1.43) | 0.066 |
| Q3 | 1.96 (1.65~2.33) | <0.001 | 1.58 (1.32~1.89) | <0.001 | 1.30 (1.06~1.58) | 0.011 |
| Q4 | 1.99 (1.68~2.36) | <0.001 | 1.53 (1.28~1.84) | <0.001 | 1.19 (0.96~1.47) | 0.121 |
| *P* for trend |  | <0.001 |  | <0.001 |  | 0.032 |

Model 1: Unadjusted.

Model 2: Adjusted for age and gender.

Model 3: Adjusted for age, gender, BMI, smoking, drinking, hypertension, diabetes, CHD, NEU, LDL-C, and HDL-C.

OR, Odds Ratio; CI, Confidence Interval; BMI, body mass index; CHD, coronary heart disease; NEU, Neutrophils count; LDL-C, low-density lipoprotein cholesterol; HDL-C, high-density lipoprotein cholesterol

**Supplementary Table S5** Heterogeneity assessment of genetic association of TyG index with colorectal polyps

| exposure | outcome | method | Q | Q_df | Q_pval |
| --- | --- | --- | --- | --- | --- |
| TyG | colon polyps | MR Egger | 280.744016475524 | 159 | < 0.001 |
| TyG | colon polyps | Inverse variance weighted | 280.781476602291 | 160 | < 0.001 |
| TyG | rectal polyps | MR Egger | 164.068617590402 | 163 | 0.461 |
| TyG | rectal polyps | Inverse variance weighted | 164.070359080539 | 164 | 0.484 |

**Supplementary Figure S1.** Overview of the study design for MR analysis exploring the causal relationship between TyG index and colorectal polyps


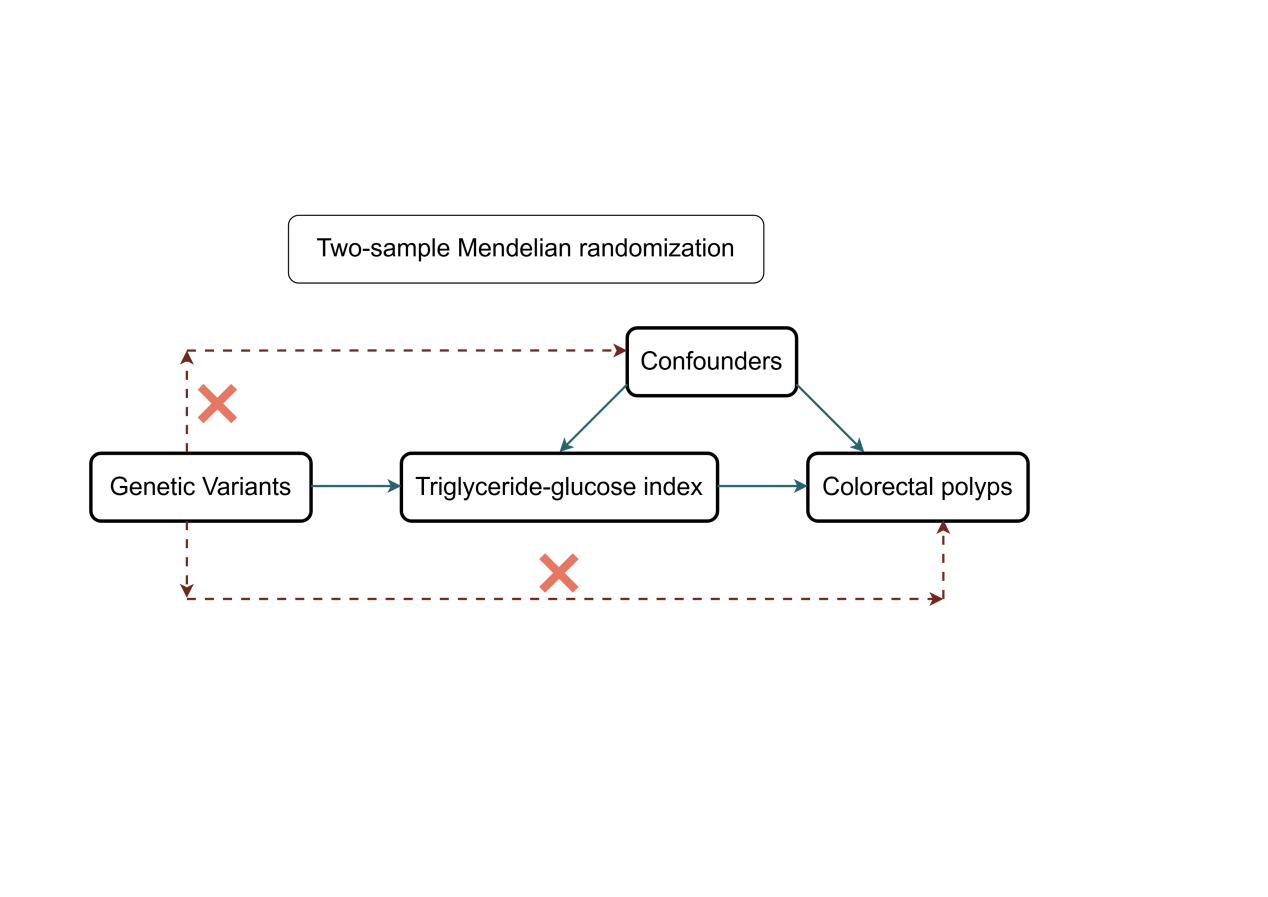


**Supplementary Figure S2.** Restricted cubic spline curve for the association between TyG index and the prevalence of colorectal polyps


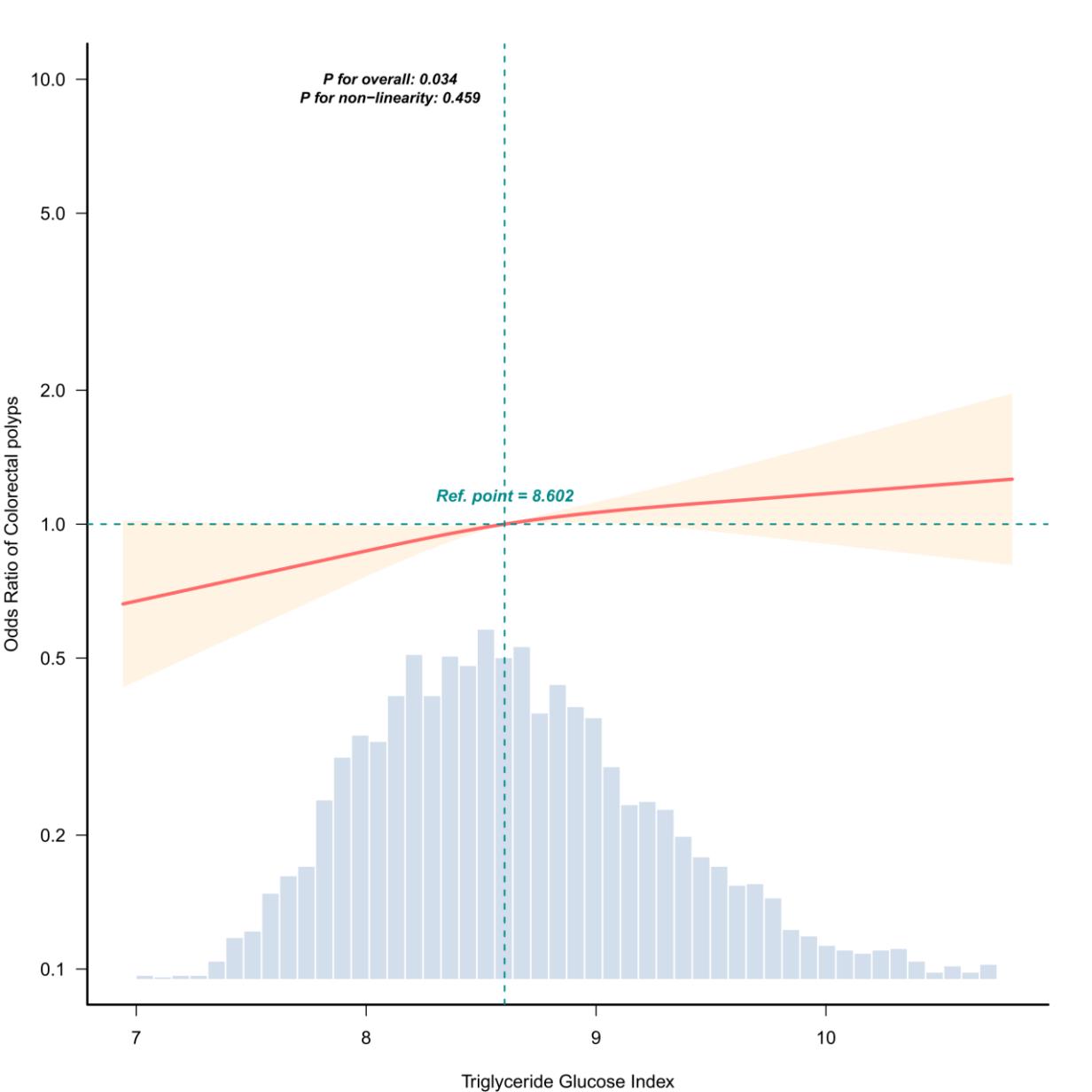


The red solid lines indicate multivariate-adjusted hazard ratios and the yellow zone indicate the 95%Cis derived from restricted cubic spline regression. The restricted cubic spline model was adjusted for age, gender, BMI, smoking, drinking, hypertension, diabetes, CHD, NEU, LDL-C, and HDL-C. Only 99%of the data is shown.

BMI, body mass index; CHD, coronary heart disease; NEU, Neutrophils count; LDL-C, low-density lipoprotein cholesterol; HDL-C, high-density lipoprotein cholesterol

**Supplementary Figure S3.** The funnel plot of genetic association of TyG index with (A) colon polyps and (B) rectal polyps.


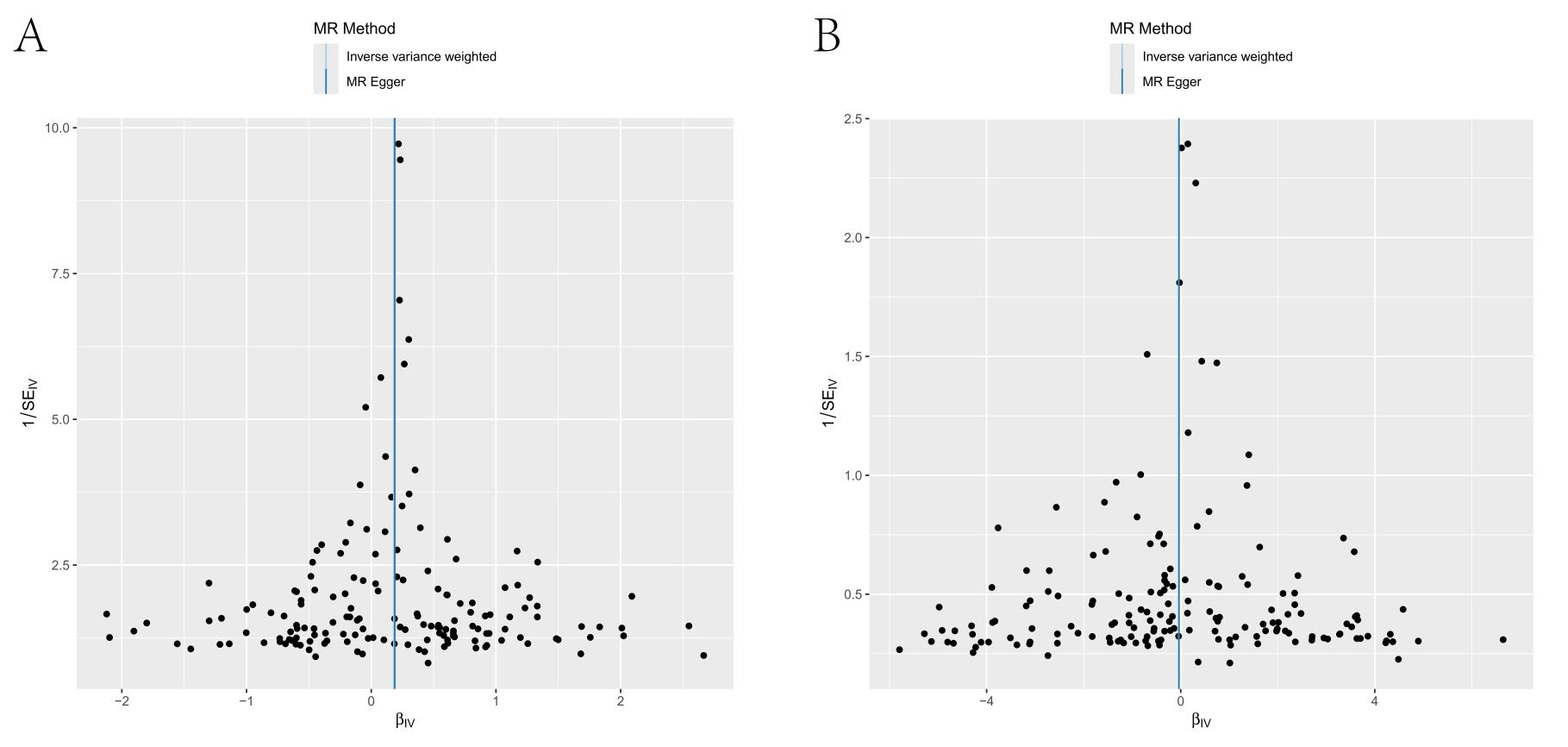

Supplement: Supplementary file 1 [file DataSheet1.docx]
